# Delafossite CuCoO_2_/ZnO derived from ZIF-8 heterojunctions as efficient photoelectrodes for dye-sensitized solar cells

**DOI:** 10.1039/d3ra01595e

**Published:** 2023-05-15

**Authors:** Mostafa Roudgar-Amoli, Amin Alizadeh, Ebrahim Abedini, Zahra Shariatinia

**Affiliations:** a Department of Chemistry, Amirkabir University of Technology (Tehran Polytechnic) P.O. Box: 15875-4413 Tehran Iran aminchemist7th@gmail.com shariati@aut.ac.ir

## Abstract

To achieve high-performance dye-sensitized solar cells (DSSCs), it is essential to establish new and effective photoelectrode materials. Herein, we report the successful synthesis of heterojunctions including Cu-based delafossite oxide CuCoO_2_ and ZnO derived from zeolitic imidazolate framework-8 (ZIF-8). The layered polyhedral nanocrystals of CuCoO_2_ produced through a feasible low temperature hydrothermal process and the faceted nanocrystals of ZnO were achieved by heat treatment of ZIF-8. The composite heterostructures were applied as photoelectrodes in DSSCs assembled using dye N719 and a Pt counter electrode. The physicochemical characteristics (XRD, FESEM, EDAX, mapping, BET, DRS), dye loading, and photovoltaic properties (*J*–*V*, EIS, IPCE) of the fabricated materials were studied and fully discussed. Results revealed that addition of CuCoO_2_ to ZnO significantly improved the *V*_oc_, *J*_sc_, PCE, FF, and IPCE. Among all cells, CuCoO_2_/ZnO (0.1 : 1) showed the best performance (PCE = 6.27%, *J*_sc_ = 14.56 mA cm^−2^, *V*_oc_ = 687.84 mV, FF = 62.67%, IPCE = 45.22%) and acted as a promising photoanode in DSSCs.

## Abstract 2

Delafossite CuCoO_2_/ZIF-8-derived ZnO heterojunctions in various ratios were used as photoelectrodes of dye-sensitized solar cells. The champion device exhibited highest PCE of 6.27%, *J*_sc_ = 14.56 mA cm^−2^, *V*_oc_ = 687.84 mV, FF = 62.67%.

## Graphical abstract

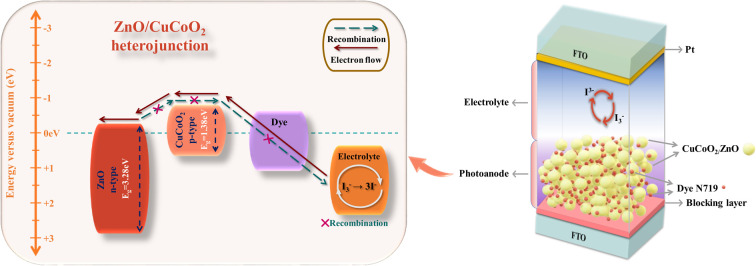

## 1. Introduction

All living things on Earth receive energy from the Sun *via* the process of photosynthesis, making it the most essential and sustainable energy source currently available.^1^ It is possible to directly convert the Sun's energy into useable heat or electricity.^2^ Therefore, scientists and engineers are attempting to create solar-powered devices that can generate electricity more efficiently.^3^ Solar radiation can be directly converted into electrical energy by a photovoltaic cell. Monocrystalline silicon solar cells are the 1st generation of solar cells.^4^ These solar cells are more efficient than conventional solar cells as they are constructed from silicon wafers.^5^ The high cost of their fabrication, however, increases the price of crystalline solar cells that are readily available on the market.^6^ The second generation of solar cells are thin film solar cells. The thin film solar materials are in powder form, which give the cell additional flexibility and lightness.^7^ The low efficiency is the key challenge facing thin film solar cells.^8^

The DSSC belongs to the third solar cell generation. These solar cells have a higher efficiency than thin film devices but a lower efficiency than crystalline solar cells.^9^ O'Regan and Gratzel created the first DSSC in 1991.^10^ The DSSC can typically create electrons by the absorption of light, transport the electrons upon application of high voltages, and then return the electrons to the device at low voltages. The photoanode working electrode, which is a mesoporous semiconductor film placed onto transparent, conductive glass (often fluorine doped thin oxide, FTO), the iodide/triiodide (I^−^/I_3_^−^) redox electrolyte, and Pt counter electrode (cathode) are typically used to produce DSSCs.^11^ One of the most important components of solar cells, the photoanode, is responsible for the charge collection and transfer.^12^

The best DSSCs combine a photoanode with a high surface area (almost 1200 times that of a flat electrode) with a dye of high extinction to provide good light gathering efficiency.^13^ The light absorption can be extended into the red wavelengths indicating this combination enables adequate absorption over most of the visible spectrum.^14^ The photons absorbed by dye molecules produce excitons that are split on a time scale of ten femtoseconds in the most effective cells, leading to charge separation efficiencies that are close to unity.^15^ The difference between the rate of charge transport and the rate of charge recombination determines that how effectively the final step (charge collecting) operates.^16^ Iodide/triiodide (I_3_^−^/I^−^) is an extremely slow redox shuttle that has to be utilized in order to compete with relatively slow (*ca.* millisecond) transport through the nanoparticle network and to prevent recombination. Because of this, DSSCs based on TiO_2_ and iodide/triiodide display excellent electron collecting while having low apparent electron diffusion coefficients.^17^

Due to the suitable surface area, durability, and electrical characteristics, photoanode materials notably ZnO, TiO_2_, and Nb_2_O_5_ are widely employed in DSSCs.^18^ ZnO is a semiconductor with a wurtzite hexagonal structure, a high melting temperature of 2248 K, and a high cohesive energy of 1.89 eV for strong bonds. It has a direct and wide band gap (*E*_g_ = 3.03 eV), extraordinary chemical stability, high exciton binding energy (60 meV), high optical gain, photo-luminescence, and piezoelectric properties, all of which make it very useful for a variety of optical devices. It is also non-toxic and abundant in nature. The inherent defects, such as O vacancies and Zn interstitials, which are present within the non-stoichiometric undoped ZnO thin films, in general display n-type electrical conductivity with very high electron densities of about 10^21^ cm^−3^.^19^ The ZnO films are highly useful in solar cells due to their combination of high electrical conductivity, good visible transmittance, and effective light scattering properties.^20^ Additionally, their pyramidal design was appropriate to utilize them as front electrodes (photoanode) and antireflective coatings in solar cells.^21^

Highly ordered and multidimensional ZnO nanostructures with hierarchical structures have been used frequently to increase the device efficiency in gas sensors, photocatalytic applications, and DSSCs.^22^ One of the best alternatives to the TiO_2_ semiconductor in DSSC manufacturing is ZnO, which has exceptional bulk electron mobility that is generally one order of magnitude better than the anatase TiO_2_.^23^ Additionally, the ZnO films have low electron trapped surface state densities, which improves cell performance.^24^ However, ZnO only responds to ultraviolet (UV) light, which contributes for less than 5% of the solar spectrum's energy, due to its large *E*_g_ = 3.03 eV.^25^ According to a paper, adding different types of carbon or nitrogen to ZnO lattices can reduce the band-gap energy, extending the absorption edge into the visible light range and allowing for the use of 42% of solar energy. Either *in situ* synthesis or post-treatment can add carbon or nitrogen to ZnO. However, post-treatment often results in random distribution throughout the profile from the surface to the center of a particle, whereas *in situ* doping typically necessitates a key synthesis environment.

**Scheme 1:**
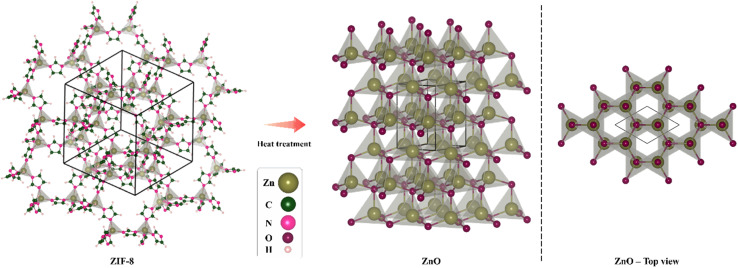
The schematic ZnO synthesis *via* heat treatment of ZIF-8. The structure of ZIF-8 is indicated in the left, the structure of ZnO obtained after heat treatment at 600 °C is visible in the middle, and the top view of the ZnO structure is seen in the right.

Metal–organic frameworks (MOFs) are solid, porous nanomaterials made of inorganic hybrid centers or metal ions connected by organic linker molecules. In addition to their direct applications, MOFs may be easily and controllably employed as sacrificial templates or precursors to generate a variety of hybrid inorganic nanomaterials.^26^ ZIF-8, a Zn-containing N-rich MOF, is composed of Zn atoms attached to MeIm, with the formula Zn(MeIm)_2_. Without the need to a stabilizing agent or activation procedures like heating, microwave irradiation, or ultrasound, it is feasible to efficiently synthesize ZIF-8.^27^ Recently, ZIF-8 was thermally treated to create porous ZnO photocatalysts, which improved methylene blue degradation.^28^ Such a method would be investigated further for hybrid synthesis and heteroatom doping.^29^ Compared to previous *in situ* or post-treatment techniques of carbon- and/or nitrogen-doping, the synthesis would be simpler. Taikei Enomoto *et al.* fabricated ZnO derived from ZIF-8 and employed it in DSSC photoanode and studied its performance.^30^ Huifen Fu *et al.* used this method to achieve N-doped ZnO and examined its capability in ethanol-sensing.^31^Scheme 1 illustrates the schematic ZnO production through heat treatment of ZIF-8. On the left side, the structure of ZIF-8 can be seen, in the middle part, the structure of ZnO obtained through heat treatment at 600 °C is displayed, and in the right side, the structure of ZnO is presented from the top view.

Researchers used a variety of approaches, including introduction of dopants, modifying size and shape of semiconductor materials, and formation of heterojunctions, to promote absorption of these semiconductors in the visible light region and to suppress the charge carrier recombination.^32–34^ Among them, creating heterojunctions between the photocatalyst materials and other semiconductor oxides would allow for increased activity by efficiently separating the charge-carriers through the creation of an interfacial electric field.^35,36^ Among numerous heterojunctions, p–n heterojunctions have demonstrated to be favorable for effective charge separation, extended lifetimes, and quick charge transfer.^37^

Due to their broad usage in optics, magnetism, catalysis, and super-capacitors, copper oxide-based p-type delafossite materials (CuMO_2_, where M is 3d metal) have recently attracted increasing attention as electrodes.^38^ In the past ten years, they have also achieved notable advancements in the conversion of solar energy, environmental protection, degradation of organic pollutants, and p-type DSSCs.^39,40^ The CuCoO_2_ is an example of copper oxide-based delafossites which has drawn particular interest due its efficiency, chemical and electrochemical stability, high catalytic activity, and superior corrosion resistance. It has been employed in a variety of applications such as photocatalysis showing good optical and photovoltaic properties.^41^ Also, hole-doped polycrystals and thermoelectric materials were used in supercapacitors.^42^ Additionally, it was utilized as a co-catalyst to increase the efficiency of charge separation and transfer in other materials, such as BiVO_4_ film for the oxygen evolution reaction.^43^ Numerous techniques including solid state reaction, sol–gel, spray pyrolysis, magnetron sputtering, hydrothermal, and more recently co-precipitation at low temperature, have been described for the synthesis of delafossites.^44^

**Scheme 2:**
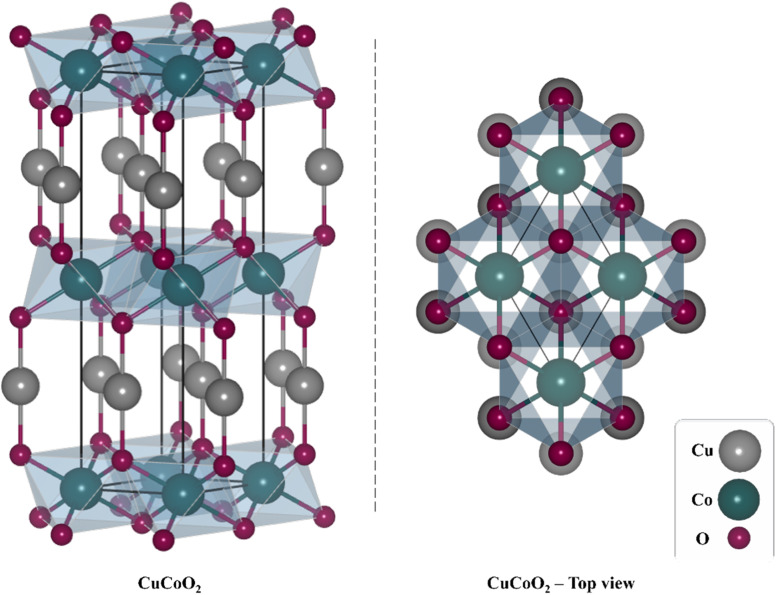
The rhombohedral crystal structure of the delafossite CuCoO_2_. A depiction of top view of the CuCoO_2_ can be seen in the left.

The rhombohedral crystal structure of CuCoO_2_ is depicted in Scheme 2. The monovalent cation Cu^+^ is linearly coordinated with oxide O^2−^ anions in the delafossite layers, whereas the trivalent cation Co^3+^ is octahedrally surrounded by six O^2−^ anions. Additionally, the O^2−^ bonds with three equivalent Co^3+^ cations and one equivalent Cu^+^ cation creates a mixture of deformed edge-corner sharing OCuCo_3_ trigonal pyramids.^45^ CuCoO_2_ delafossite in particular can function as a potential p-type semiconductor for increasing the activity of DSSC due to its absorption in the visible region with a small bandgap energy of about 1.60 eV. However, because of the greater rate of electron–hole recombination, it has poor transport characteristics.^38^

**Scheme 3:**
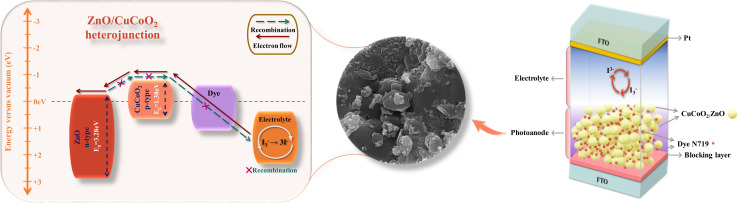
The fabrication of the dye-sensitized solar cell. On the right side, a DSSC can be seen, in which the photoanode is composed of the CuCoO_2_/ZnO composite shown in the form of pale-yellow circles along with N719 dye molecules (red circles). In the middle part, the FESEM micrograph of the composite photoanode is observed. On the left side, the band diagram has been exhibited for the heterostructure containing CuCoO_2_ and ZnO (band gaps are taken from Tauc plots); including the electron path through the photoanode and the inhibition of recombination.

To overcome these difficulties, we present the development of heterostructures between ZnO as n-type wide bandgap semiconductor and CuCoO_2_ as p-type narrow bandgap semiconductor for increasing the open-circuit voltage (*V*_oc_) and enhancing photoelectric conversion efficiency (PCE) of DSSCs.^46^ In this work, ZnO nanoparticles were successfully synthesized through a thermal treatment process of ZIF-8 as a self-sacrificial template. N atoms were doped into the lattice of ZnO nanoparticles using this procedure. The copper oxide-based delafossite CuCoO_2_ nanoparticles were also synthesized through a simple, low temperature hydrothermal method. Through combination of the mentioned materials, the CuCoO_2_/ZnO composites were produced with different mole ratios of CuCoO_2_ to ZnO (0.05, 0.1, 0.15, 0.2) : 1. The composites were studied in DSSCs as photoelectrodes. The experimental results showed that CuCoO_2_/ZnO composite could serve in a promising way to enhance DSSC performance. The fabrication of CuCoO_2_/ZnO composite photoanode and its corresponding band diagram formed by CuCoO_2_ (ref. 45) and ZnO^47^ is depicted in Scheme 3. As it is illustrated in this diagram, the electrons excited to the N719 dye's conduction band are then moved to the CuCoO_2_/ZnO composite's conduction band, where they eventually find their way to the FTO and external circuit. The higher level of the conduction band of CuCoO_2_ compound after forming heterostructure with ZnO prevents the return of electrons from the conduction band of ZnO to CuCoO_2_. Scheme 3 also depicts the CuCoO_2_/ZnO as a single layered photoanode that completely contacts the redox electrolyte, demonstrating how recombination is inhibited.^48^

## 2. Experimental

### 2.1. Materials

Zn(CH_3_COO)_2_·2H_2_O, 2-methylimidazole (2-MeIm), Co(NO_3_)_2_·6H_2_O, Cu(NO_3_)_2_·3H_2_O, ethyl cellulose, terpineol (C_10_H_18_O), acetone, acetonitrile, ethanol, 2-propanol, I_2_, LiI, SiO_2_, HCl, 4-*tert*-butyl pyridine, titanium(iv) tetra-isopropoxide (Ti[OCH(CH_3_)_2_]_4_), H_2_PtCl_6_–6H_2_O, and N719 dye were provided by the Sigma-Aldrich and Merck Companies and used exactly as they were delivered. Solaronix Company provided the Surlyn spacer and conductive FTO (80% transparency in visible region, ∼30 Ω cm^−2^) coated glasses.

### 2.2. ZIF-8 synthesis

**Fig. 1:**
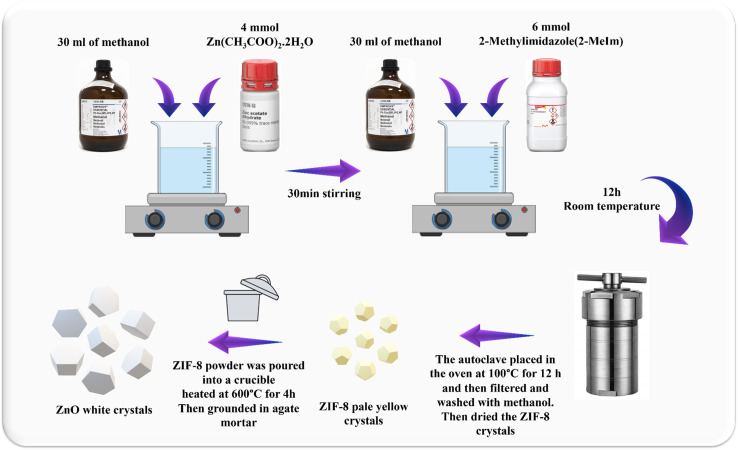
Schematic illustration of synthesis of the hexagonal wurtzite ZnO nanocrystals derived from ZIF-8 through heat treatment at 600 °C.

4 mmol of Zn(CH_3_COO)_2_·2H_2_O and 6 mmol of 2-MeIm were weighed and dissolved separately in 30 mL of methanol. After 12 hours under stirring at room temperature, the solution was transferred to the autoclave and heated in the oven at 100 °C for 12 hours. Next, the pale-yellow crystals of ZIF-8 were filtered, washed three times with methanol and dried to obtain the solid powder (Fig. 1).

### 2.3. ZnO synthesis

The synthesized ZIF-8 powder was poured into a crucible and heated at 600 °C for 4 hours under air flow with heating rate of 2.5 °C min^−1^. After cooling down to room temperature, the crystals were slightly grounded in an agate mortar. The obtained product was white colored powder of ZnO (Fig. 1).

### 2.4. CuCoO_2_ synthesis

**Fig. 2:**
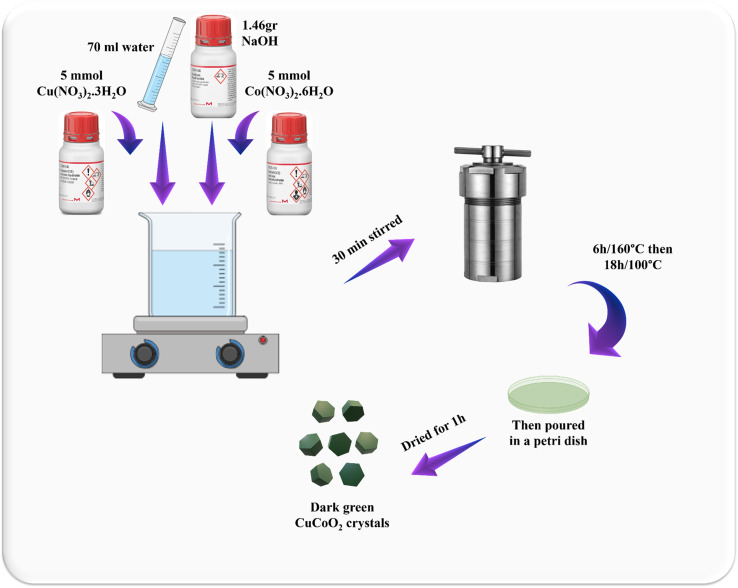
Schematic illustration of hydrothermal synthesis of the delafossite CuCoO_2_.

5 mmol of Co(NO_3_)_2_·6H_2_O and 5 mmol of Cu(NO_3_)_2_·3H_2_O were respectively dissolved in 70 mL of water. Then, a certain amount of NaOH (1.46 g) was weighed and added to the solution. After half an hour, the solution was poured into an autoclave. It was heated in the oven for 6 hours at 160 °C and then at 100 °C for 18 hours. Subsequently, it was dried in a Petri dish for an hour and a dark green powder was obtained (Fig. 2).

### 2.5. CuCoO_2_/ZnO composites synthesis

**Fig. 3:**
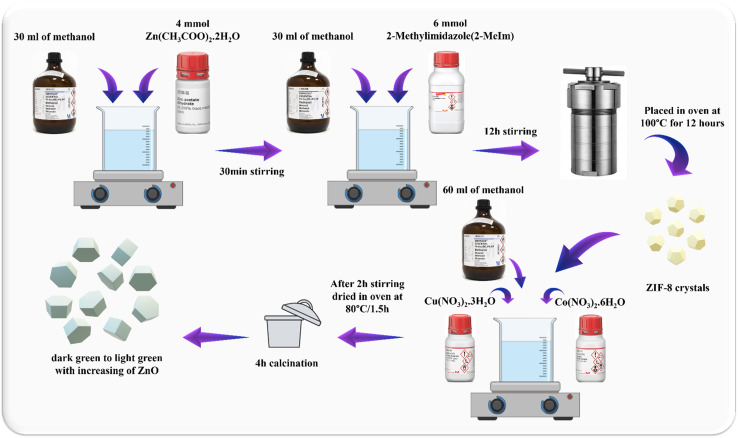
Schematic illustration of the synthesis of CuCoO_2_/ZnO composite.

The CuCoO_2_/ZnO composites were made by adding delafossite precursors to the solution containing ZnO crystals and then calcination at 600 °C. The process was such that 8 mmol of Zn(CH_3_COO)_2_·2H_2_O and 12 mmol of 2-MeIm were separately dissolved in 50 mL methanol. After 12 hours under stirring at room temperature, the solution was transferred to the autoclave and heated in oven at 100 °C for 12 hours. Next, delafossite precursors (Co(NO_3_)_2_·6H_2_O and Cu(NO_3_)_2_·3H_2_O) were added to a certain molar ratio of zinc to synthesize the CuCoO_2_/ZnO composites in (0.05, 0.1, 0.15, 0.2) : 1 ratios. The dried ZIF-8 crystals and these precursors were dispersed in 60 mL methanol and stirred for 2 hours. Subsequently, the solution was dried in oven at 80 °C for 1.5 h and the obtained powder was moved into a crucible. The crucible was heated under air flow for 200 minutes at a temperature of 500 °C with a heating rate of approximately 2.5 °C min^−1^. The temperature was then raised to 600 °C at a heating rate of 5 °C min^−1^, held there for 20 minutes, and then cooled to 25 °C in the open air. The resulting materials' color changed from dark green to light green with increasing zinc oxide content (Fig. 3).

### 2.6. Preparation of pastes

Production of paste for each nanomaterial, including pure ZnO, CuCoO_2_, and CuCoO_2_/ZnO composites in (0.05, 0.1, 0.15, 0.2) : 1 mole ratios, was done separately. For this purpose, we vigorously mixed 0.2 g of ethyl cellulose, 0.8 g of terpineol and 2 mL anhydrous ethanol to obtain a viscous white solution. The viscous solution was stirred for 12 hours at 25 °C and then we added 0.250 mg of each nanomaterial into the vessel. The mixtures were thoroughly stirred on a magnetic stirrer for an additional 4 h to attain homogeneous pastes.

### 2.7. DSSCs assembly

**Fig. 4:**
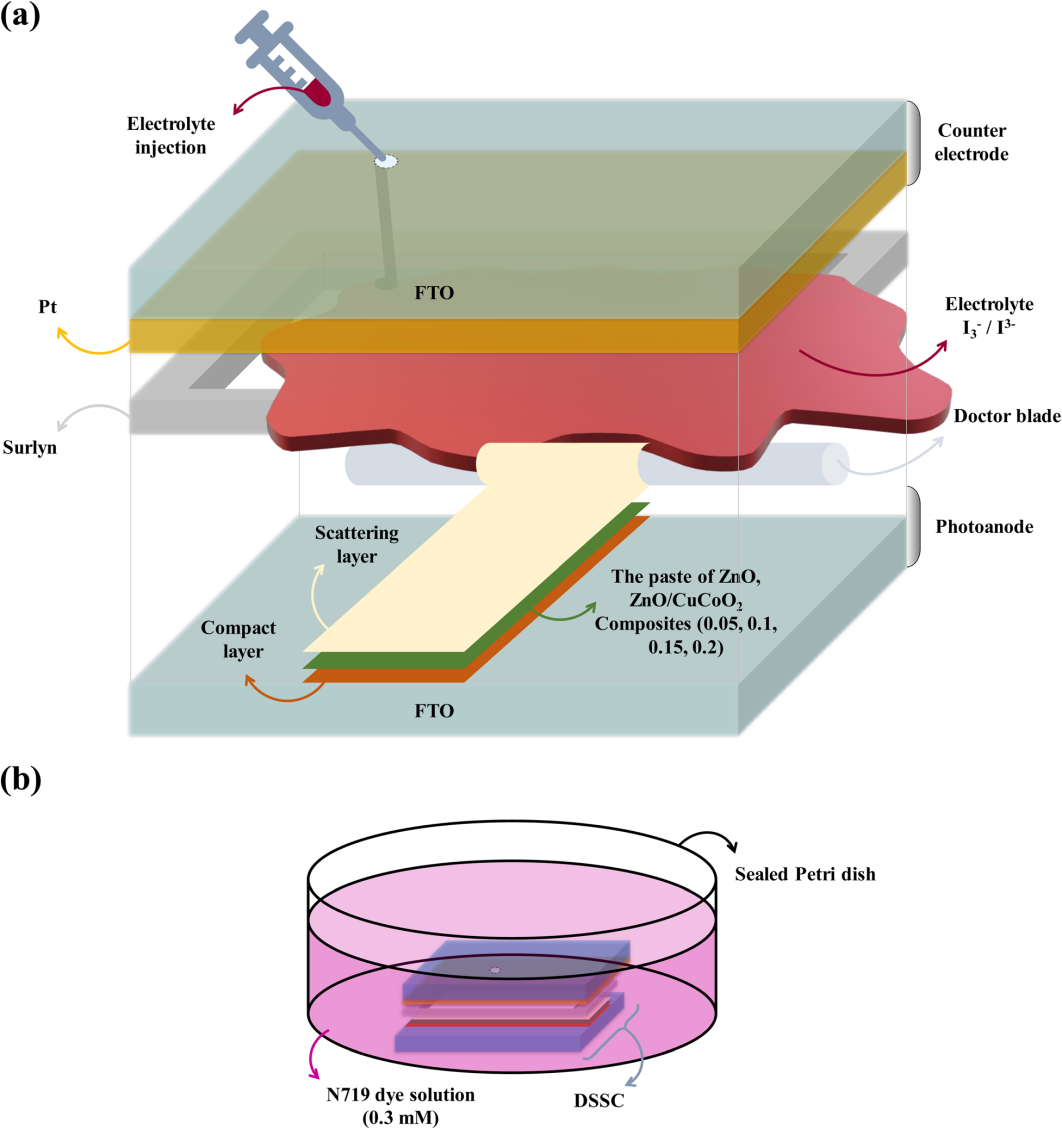
Schematic illustration of (a) fabrication of a DSSC composed of photoanode (FTO, compact layer, paste of the synthesized materials, scattering layer), Surlyn, electrolyte, and counter electrode (Pt/FTO). (b) Soaking of the fabricated DSSC in N719 dye solution.

As it is illustrated in Fig. 4, two transparent, highly conductive substrate sheets that offer a surface for depositing semiconductor electrodes are commonly used to assemble a DSSC. Sunlight can enter the cell's active region directly through a highly transparent substrate. To achieve the optimum results and light absorption, the substrate transparency should be greater than 80%. The FTO glasses (1.44 × 1.44 cm^2^) were cleaned using detergent, water, HCl (0.1 M), acetone, and ethanol, in that order. Each washing procedure was performed for 10 minutes at 75 °C in an ultrasonic device. Then, FTO glasses were dried in oven. They were employed as counter electrodes and photoanodes after drying. To create a photoanode, the FTO substrate was coated with two kinds of pastes, including a compact TiO_2_ paste (c-TiO_2_), and the scattering paste. The solution for the c-TiO_2_ layer contains 0.37 mL Ti[OCH(CH_3_)_2_]_4_ in ethanol (5 mL) and a drop of HCl, which was spin-coated onto FTO (2000 rpm, 30 s). The material was heated for 10 minutes at 80 °C in oven following the c-TiO_2_ deposition, and finally it was annealed for 30 minutes at 500 °C. After that, using the doctor blade technique, several pastes including pure ZnO, CuCoO_2_, and composites CuCoO_2_/ZnO in (0.05, 0.1, 0.15, 0.2) : 1 ratios were coated on the compact TiO_2_ layer. The slurries were well combined and heated to 35 °C for 15 minutes. The coated FTO glasses were subsequently heated and dried in oven at 110 °C for 15 minutes, and they were annealed at 500 °C for 30 minutes. The light scattering layer was applied on top of the second layer using the doctor blade method as the last stage, and it was dried and calcined for 30 minutes at 500 °C (Fig. 4a). TiO_2_, ethyl cellulose, and SiO_2_ were blended to achieve a viscous, transparent solution to create TiO_2_ scattering paste. The TiO_2_ film was sensitized until maximal dye adsorption occurred when the photoanode was soaked in N719 dye solution (0.3 mM) for 20 hours (Fig. 4b). The dye-sensitized films were then cleaned with ethanol to wash extra dye molecules. FTO glass was coated with a 0.5 mM H_2_PtCl_6_ solution in 2-propanol to create the counter electrode. The counter electrode was then subjected to a 20 min treatment at 450 °C. In the acetonitrile-based electrolyte (1 mL) of DSSCs, 4-*tert*-butyl pyridine (0.5 M), iodine (I_2_) (0.05 M), and lithium iodide (LiI) (0.1 M) were present. At 120 °C, the counter electrode and photoanode were connected, and the Surlyn spacer was used to complete the sandwich-type cell. To stop solution vaporization, electrolyte was injected into the internal area of each predrilled FTO before it was sealed.

### 2.8. Analysis methods

A field emission scanning electron microscope (FE-SEM, TESCAN MIRA-3) was used to analyze particle sizes and morphologies. Energy-dispersive X-ray spectroscopy (EDX, Oxford Instrument, and England) was employed to produce elemental analyses and mapping images. Results from X-ray diffraction (XRD) were used to identify each sample's crystallinity and crystalline phase using the INEL EQUINOX 3000 X-ray diffractometer at 30 kV and 30 mA with a Cu-K wavelength of 0.15406 nm in the 5–100° range. Each XRD pattern was examined using the X'Pert High Score program. UV-visible reflectance characteristics are determined by the UV-visible diffuse reflectance spectrum (UV-Vis DRS) (Avaspec-2048-TEC). The volume of pores, average pore diameters, and specific surface areas of samples were all calculated using volumetric adsorption analyzers (Belsorp mini and Finetec). Under high pressure, liquid nitrogen was used to calculate the total volume of pores. Electrochemical impedance spectra (EIS) and current density–voltage (*J*–*V*) diagrams were analyzed under illumination by the electrochemical analyzer potentiostat galvanostat (OrigaLys). 100 mW cm^−2^ or AM 1.5 of simulated sunlight was produced by a solar simulator. With the use of an incident photon-to-current conversion efficiency (IPCE) spectrometer (PVE 300), IPCE spectra were aquired.

## 3. Results and discussion

### 3.1. Structural analysis

**Fig. 5:**
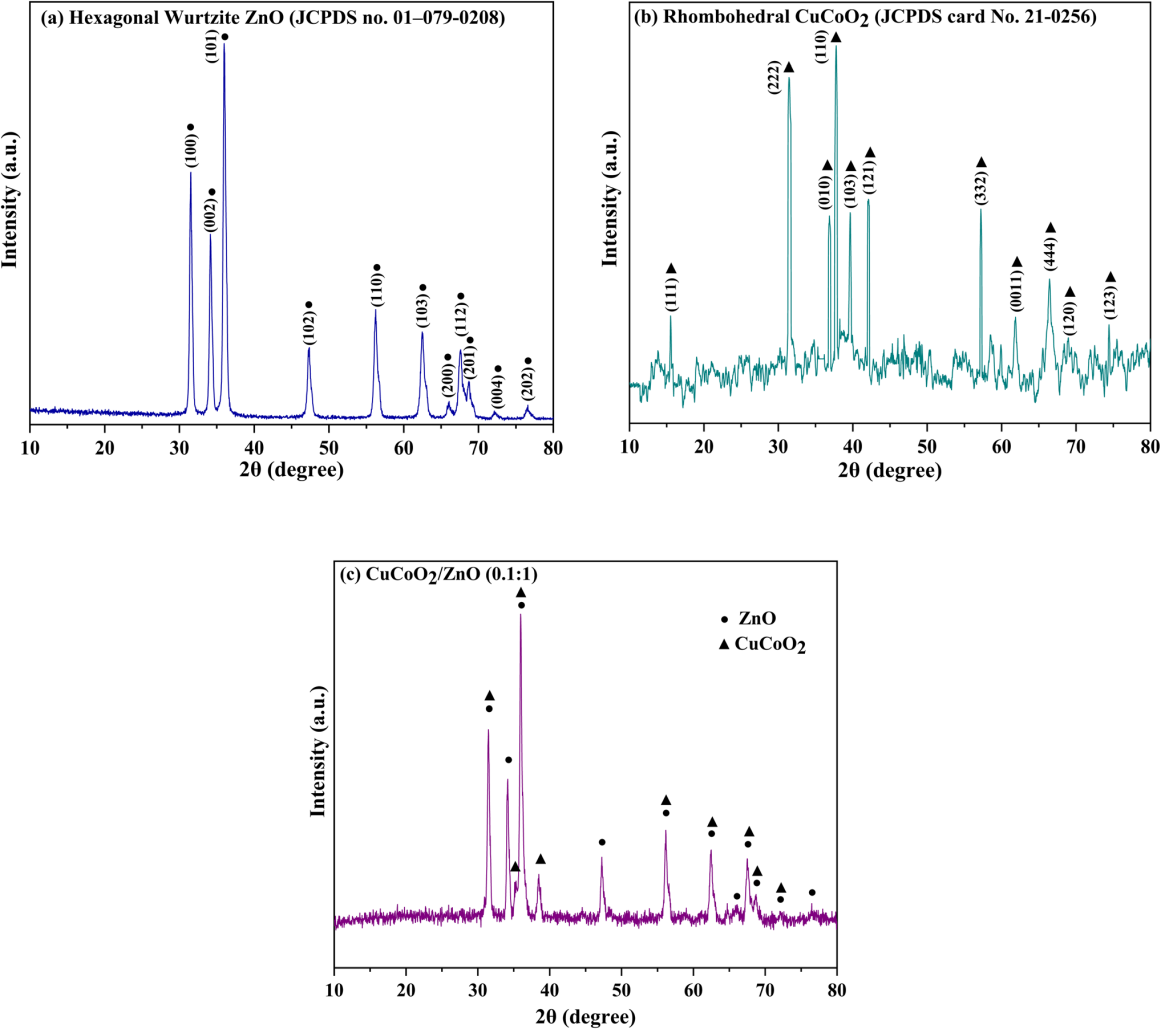
XRD patterns of (a) ZnO, (b) CuCoO_2_, and (c) CuCoO_2_/ZnO (0.1 : 1).

The crystal structures of the pure ZnO, CuCoO_2_ and CuCoO_2_/ZnO (0.1 : 1) were investigated by XRD analysis (Fig. 5). Fig. 5a reveals the XRD pattern of ZnO derived from ZIF-8 that corresponds to wurtzite hexagonal structure (*P*6_3_*mc*, JCPDS card No. 01-079-0208 (ref. 49)). The characteristic peaks at 2*θ* values of 31.56°, 34.21°, 36.06°, 47.36°, 56.30°, 62.55°, 66.06°, 67.58°, 68.68°, 72.24° and 76.62° can be ascribed to (100), (002), (101), (102), (110), (103), (200), (112), (201), (004) and (202) crystal planes of ZnO, respectively.^50^ The XRD pattern of rhombohedral CuCoO_2_ (*R*3̄*m*, JCPDS card No. 21-0256 (ref. 51)) is observed in Fig. 5b. As can be seen, the characteristic diffraction peaks are located at angles of 15.5°, 31.5°, 36.9°, 37.7°, 39.6°, 42.1°, 57.2°, 61.8°, 65.1°, 66.8° and 74.2° which are ascribed to Miller indices (111), (222), (010), (110), (103), (121), (332), (0011), (444), (120) and (123) respectively, and no impurity phase is detected.^45^ Furthermore, the XRD peaks of CuCoO_2_/ZnO (0.1 : 1) composite are illustrated in Fig. 5c which are appeared at 31.51°, 34.18°, 35.23°, 36.01°, 38.51°, 47.29°, 56.19°, 62.47°, 64.75°, 65.94°, 67.55°, 68.63°, 72.16° and 76.56°. In the composite, there is an overlap between peaks of ZnO (at 31.56°) and CuCoO_2_ (at 31.5°), which has led to the creation of a sharper peak. The peak at 37.7° for CuCoO_2_ with a small amount of shift at 38.51° can be seen in the composite. Also, the small shoulder in the peak at 35.6° for composite is related to the peak at 36.9° from CuCoO_2_. In general, the peaks of the components can be detected inside the XRD of the composite. The XRD peaks of the composite have a very slight shift compared to the peaks of the components, which indicates the presence of both components inside the composite without changing their crystal phases. Due to the low content of delafossite material in the CuCoO_2_/ZnO (0.1 : 1) composite, the intensity of the main peaks of delafossite in the composite is weak. Moreover, some 2*θ* values of delafossite peaks are close to those of zinc oxide in CuCoO_2_/ZnO (0.1 : 1) composite, which may lead to the overlapped XRD peaks of delafossite and ZnO.

Scherrer equation (*D* = *kλ*/*β* cos *θ*) was used to compute the average crystallite sizes of all nanomaterials,^52,53^ where *K* = 0.89 is a constant, λ represents Cu Kα = 0.15418 nm, *θ* demonstrates the diffraction angle, and β displays the full-width half-maximum (FWHM) (in radians), respectively.^54^ The corresponding data are given in Table 1.

**Table 1:** Physical parameters of the photoanode materials

Parameter	Photoanode material		
	ZnO	CuCoO_2_	CuCoO_2_/ZnO (0.1 : 1)
Crystallite size (*D*_XRD_)/nm	21.33	18.97	26.16
Specific surface area (*S*_BET_)/m^2^ g^−1^	43.87	11.03	70.32
Pore volume (*V*_p_)/cm^3^ g^−1^	0.14	0.0086	0.56
Pore diameter (*D*_p_)/Å	23.68	18.57	24.02
Bandgap energy (*E*_g_)/eV	3.03	1.60	2.51

### 3.2. Morphological analysis

**Fig. 6:**
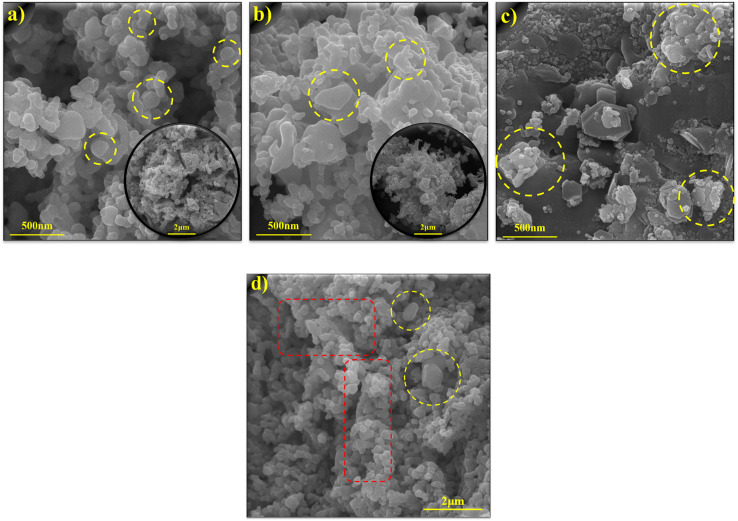
FESEM images of (a) ZnO at 500 nm (inset: 2 μm), (b) CuCoO_2_ at 500 nm (inset: 2 μm), and (c) CuCoO_2_/ZnO (0.1 : 1) at 500 nm, (d) CuCoO_2_/ZnO (0.1 : 1) at 2 μm.

The morphology and microstructure of ZnO, CuCoO_2_, and CuCoO_2_/ZnO (0.1 : 1) composite samples were investigated through FESEM images. Fig. 6a shows that the ZnO sample consists of faceted crystals that are uniformly formed with high porosity, which can be due to its synthetic route from ZIF-8 heat treatment.^55^ In Fig. 6b, layered polyhedral crystals of pure CuCoO_2_ sample are observed. Fig. 6c displays the FESEM micrograph of composite CuCoO_2_/ZnO (0.1 : 1) which signifies fabrication of uniform nanoheterojunction, where the zinc oxide and delafossite crystals are in close contact with each other on the surface. As it is depicted, we can see agglomerated particles in some places, but overall, we see high porosity in the composite.

For further investigation of the porosity of the composite sample, FESEM image was taken at another angle with a magnification of 2 μm (Fig. 6d). As we can see in the FESEM image, the porosity of the final sample increases with the determined heating rate that takes place during the composite preparation process (the distances between the particles define the porosity of the composite). Also, the yellow circles represent the delafossite but the red rectangles show a mixture of small spherical zinc oxide and delafossite, which form a high porosity structure for the composite (the increase in porosity was also determined by the BET test).

**Fig. 7:**
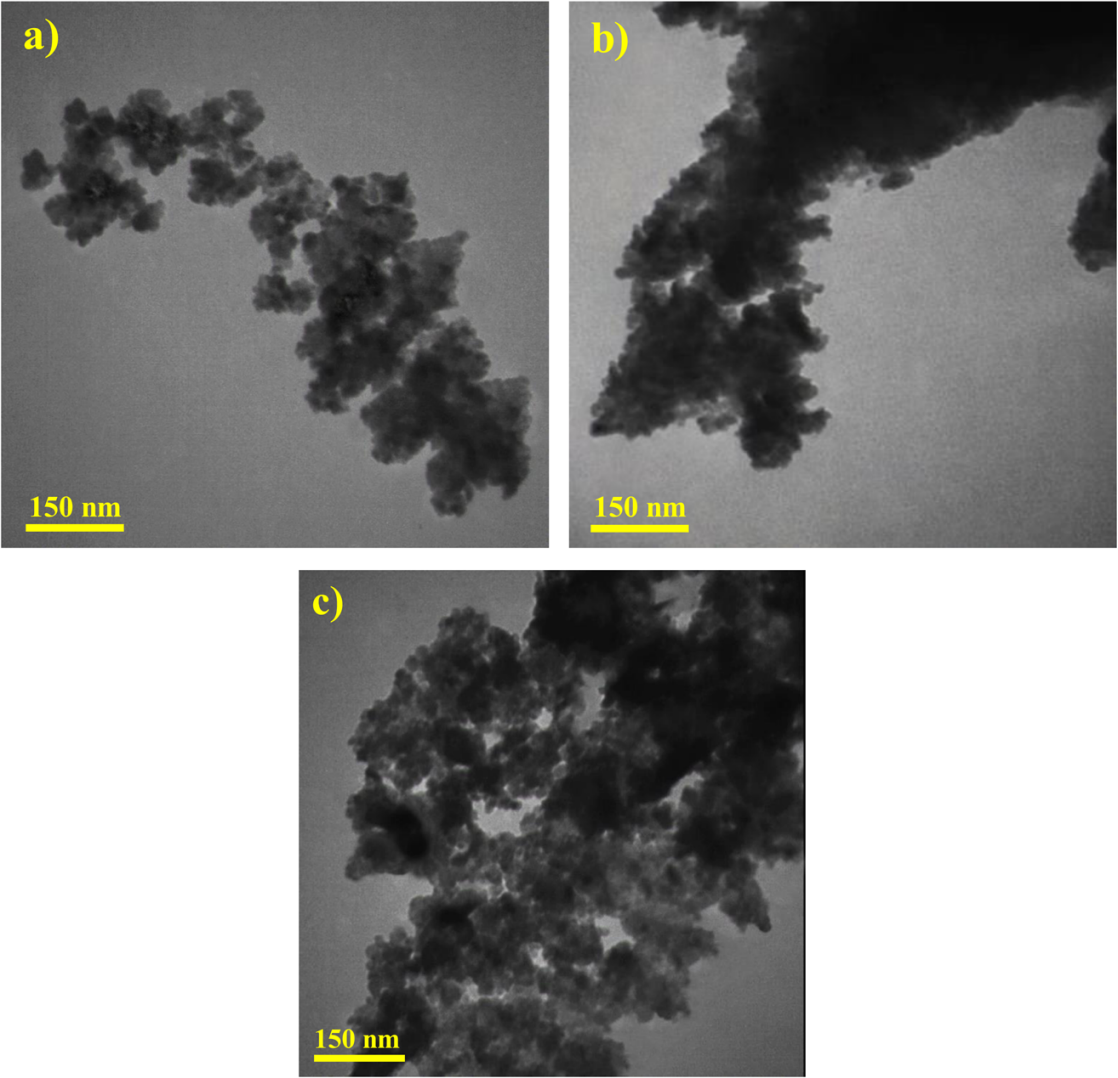
TEM images of (a) ZnO, (b) CuCoO_2_, and (c) CuCoO_2_/ZnO (0.1 : 1).

TEM images have been obtained to further explore the heterojunction structure formed between ZnO and CuCoO_2_ delafossite (see Fig. 7a–c). As can be seen in Fig. 7a, the zinc oxide has a uniform structure, uniform particle dispersion, and suitable porosity. According to TEM image in Fig. 7b, delafossite nanoparticle indicates an agglomerated structure with polyhedron form. After adding the delafossite sample with a rigid and integrated structure and performing heat treatment, the final composite has an evident porosity (Fig. 7c). Also, the presence of delafossite polyhedron can be seen next to porous zinc oxide particles, confirming they have formed a heterojunction with each other.

**Fig. 8:**
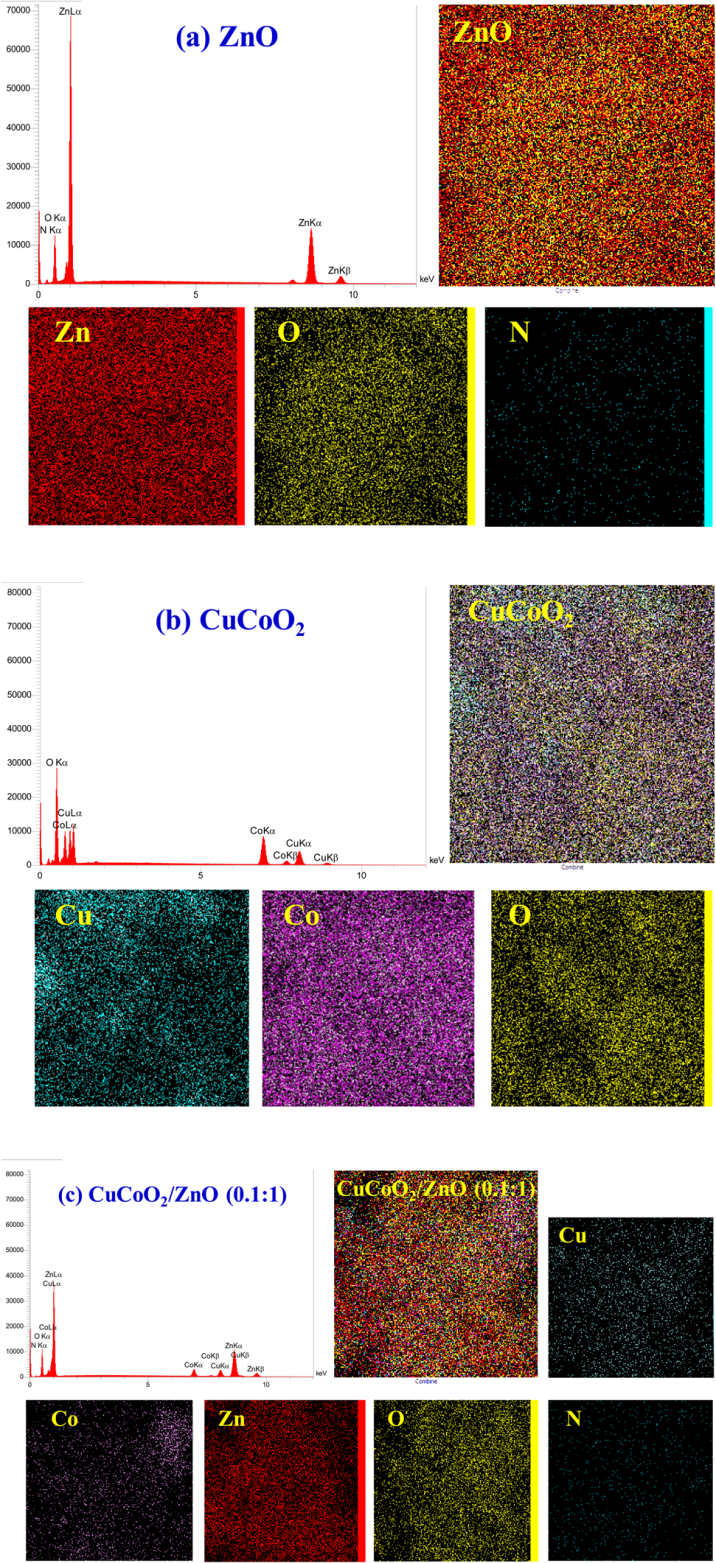
EDX and mapping analysis of (a) ZnO, (b) CuCoO_2_, and (c) CuCoO_2_/ZnO (0.1 : 1).

Fig. 8a–c show the EDX spectra correlated to the elemental compositions for ZnO, and CuCoO_2_ nanocrystals, and their nanocomposite CuCoO_2_/ZnO (0.1 : 1). Also, to investigate the element distribution in the samples, elemental mapping of a selected area is taken. It is evident that the ratio of elements is appeared as expected for ZnO nanoparticles, with the presence of a small amount of nitrogen remained in the structure after heat treatment process of ZIF-8. Existence of zinc and oxygen is evidenced too. EDX spectrum of pure CuCoO_2_ sample exhibits the presence of three elements (copper, cobalt and oxygen). The EDX of the composite CuCoO_2_/ZnO (0.1 : 1) confirms the presence of all of its constituent elements (Cu, Co, Zn, O, N).

### 3.3. Textural property

**Fig. 9:**
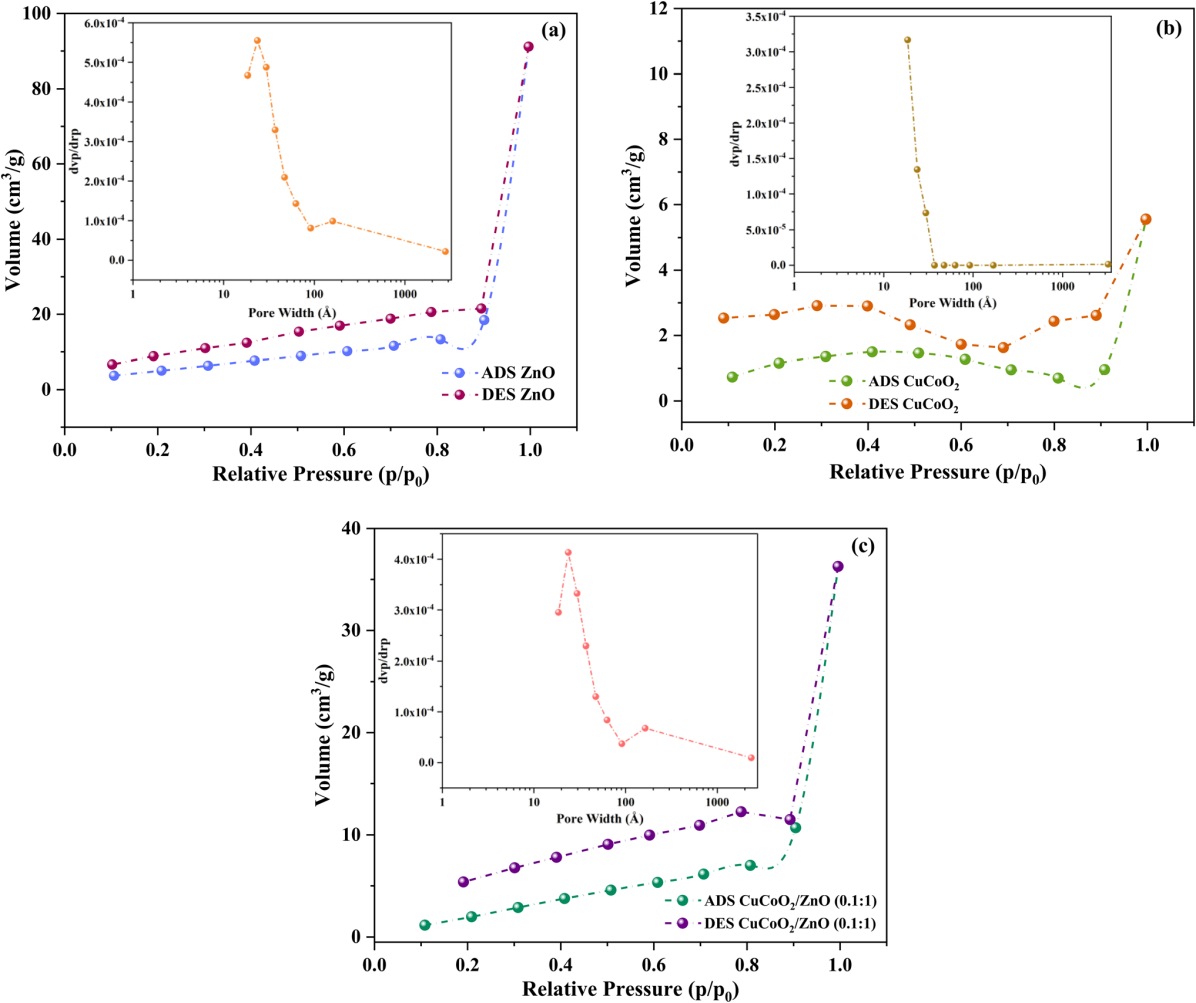
N_2_ adsorption desorption isotherms of (a) ZnO, (b) CuCoO_2_, and (c) CuCoO_2_/ZnO (0.1 : 1) (insets are the corresponding BJH pore size distributions).

One of the most important characteristics of a nanomaterial for the use in electrochemical investigations is having a large surface area. The specific surface area and pore volume of synthesized ZnO, CuCoO_2_ and CuCoO_2_/ZnO (0.1 : 1) samples were estimated using BET analysis. The N_2_ adsorption–desorption isotherms for ZnO, CuCoO_2_ and CuCoO_2_/ZnO (0.1 : 1) nanocomposite along with the corresponding BJH (Barrett–Joyner–Halenda) pore size distributions are shown in Fig. 9. Adsorption–desorption graphs demonstrate that all of them belong to the typical type IV isotherm with H3 hysteresis on the basis of IUPAC classification.^56^ The isotherms also demonstrate that the textures of produced samples are mesoporous. Mesoporous materials with high surface areas are anticipated to offer more accessible electrocatalytic active sites and effective channels for electron transfer during electrocatalytic activity in the DSSC photoanode. For samples ZnO, CuCoO_2_ and CuCoO_2_/ZnO (0.1 : 1), the obtained BET specific surface areas are 43.87, 11.03, and 70.32 m^2^ g^−1^, respectively.

According to BJH, the pore diameter based on the sharpest adsorption peak for the samples are obtained to be 23.68, 18.57, and 24.02 Å for the ZnO, CuCoO_2_, and CuCoO_2_/ZnO (0.1 : 1), respectively. This result verifies that addition of the CuCoO_2_ nanoparticles to ZnO increases the pore diameter of the CuCoO_2_/ZnO (0.1 : 1) composite nanostructure (Fig. 9, insets). For samples ZnO, CuCoO_2_ and CuCoO_2_/ZnO (0.1 : 1), the BJH pore volumes are 0.14, 0.0086, and 0.56 cm^3^ g^−1^, respectively, and the data are listed in Table 1. In general, it can be said that the composite fabrication boosts the surface area, pore diameter and pore volume. This leads to an increase in active sites along with an increase in dye loading, resulting in improved performance.

### 3.4. Optical properties

#### 3.4.1. UV-Vis DRS spectra

**Fig. 10:**
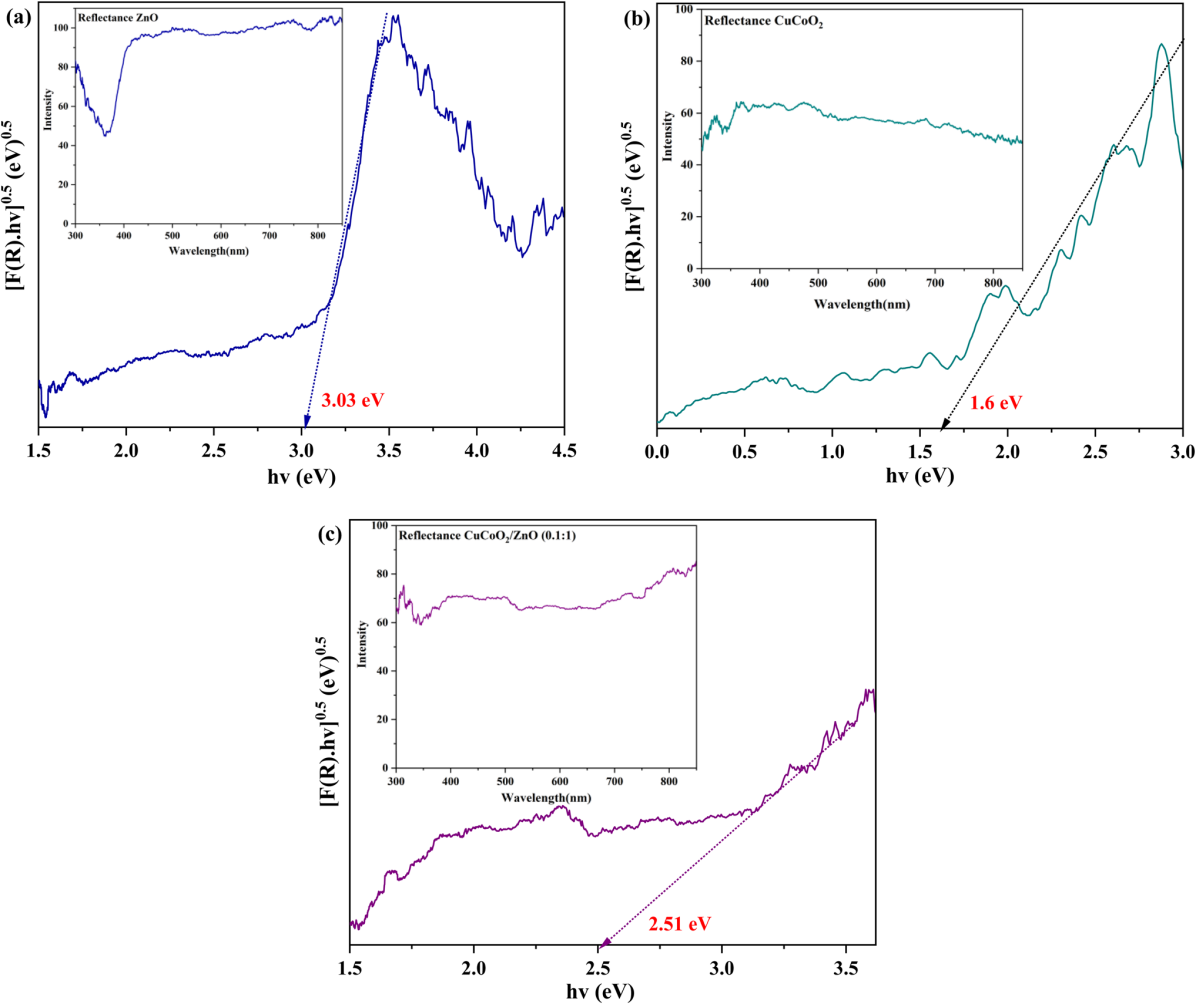
DRS plots of (a) ZnO, (b) CuCoO_2_, and (c) CuCoO_2_/ZnO (0.1 : 1). The insets are the corresponding reflectance plots.

The DRS spectra evaluate the significance of the light scattering effect in enhancing the efficiency of DSSCs.^57^ For strong light scattering, the sizes of the scattering particles must be large enough. Moreover, the structure's empty spaces result in many light reflections that significantly increase scattering and subsequently light absorption by the photoelectrode material.^58^ As a result, adding the delafossite CuCoO_2_ nanoparticles with good electronic properties (low band gap) to layered ZnO nanocrystals will improve the amount of light harvesting and light absorption at 400 nm in the composite. On the other hand, above 400 nm, the scattering influence and also the inherent properties of nanoparticles lead to an increase in light absorption by the composite (Fig. 10, insets). The optical bandgaps of both materials are ascertained from the absorption spectra using the Mott and Davis formula: (*αhν*)^*n*^ = (*hν* − *E*_g_), where *h* is Planck's constant, *α* is the optical absorption coefficient, and *ν* is the radiation frequency. The value of *n* depends on the type of transition that results in the absorption, and *E*_g_ is the optical energy gap.^48^ In our case, the value of *n* is 1/2 because CuCoO_2_ and ZnO NPs are the indirect band gap semiconducting materials, in which the transition is carried out *via* an authorized direct energy level.^59^ Tauc plot is (*αhν*)^1/2^ as a function of photon energy *hν*, which is drawn in Fig. 10.

Optical energy gaps are measured through linear fitting and extrapolation of the main absorption peak towards the *x*-axis for all samples. The *E*_g_ values of 1.60, 3.03, and 2.51 eV are obtained for pure CuCoO_2_, ZnO, and CuCoO_2_/ZnO (0.1 : 1), respectively. These data are shown in Table 1, and the energy gap of ZnO is reduced as a result of CuCoO_2_ addition into the ZnO film. This is because formation of composite alters the sample's size distribution. The wavelength range of light absorption and the capacity of CuCoO_2_ composites to absorb light are increased by its optimal band gap. Consequently, an optimal content of CuCoO_2_ enhances the light absorption property. CuCoO_2_/ZnO (0.1 : 1) has the highest light absorption and the lowest optical band gap energy, implying it may have the highest photovoltaic efficiency.

#### 3.4.2. Dye loading

**Fig. 11:**
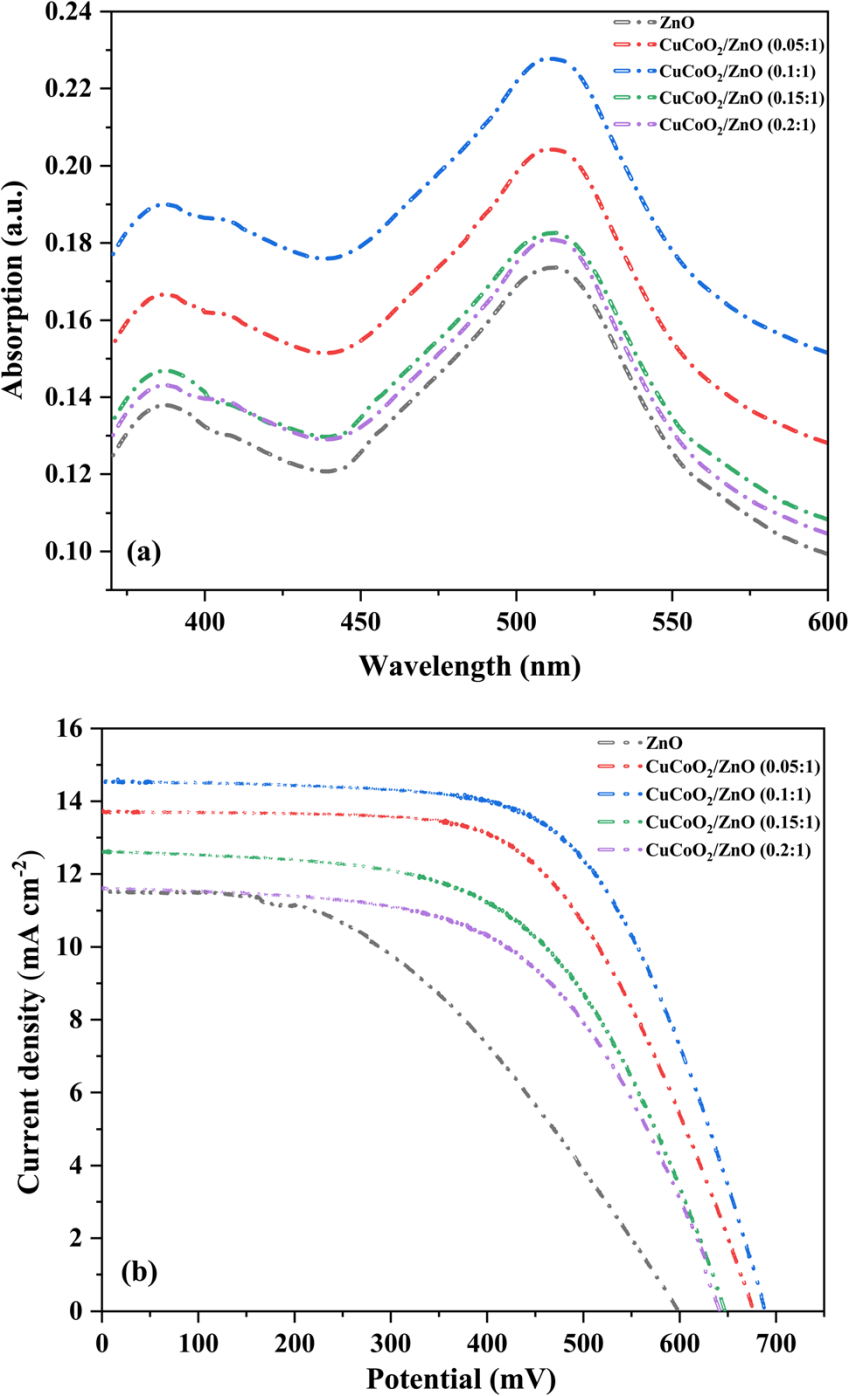
(a) Dye loading capacities of all photoanodes using UV-Vis spectra after desorption of dye molecules from the electrodes within 0.1 M NaOH in water/ethanol solution. (b) Current density–voltage (*J*–*V*) curves of the fabricated DSSCs under AM 1.5 illumination (100 mW cm^−2^).

The fundamental reason for the *J*_sc_ change is increasing the amount of dye adsorption. Using UV-Vis spectra, following the desorption of dye molecules from the electrodes inside 0.1 M NaOH in water/ethanol solution with a molar ratio of 1 : 1, we evaluated the dye loading capabilities of each photoanode. Also, the UV-Vis peak intensity for the N719 dye at 515 nm is compared to assess the relative dye adsorption of each sample. Fig. 11a clearly demonstrates that how adding the ideal quantity of CuCoO_2_ to the ZnO layer improves dye adsorption. The reason for increasing the amount of loaded dye as the CuCoO_2_ dosage is raised may be explained by the fact that the density of the nanocomposite layer decreases as nanoparticles become looser with the addition of the CuCoO_2_ nanoparticles while the bare ZnO layer exhibits a little larger density. This results in more dye molecules being adsorbed by semiconductor nanocomposites as there are greater gaps between the particles. Dye molecules may be uniformly distributed across the surface of the nanoparticle or may congregate in islands on particular crystallographic faces, leaving other faces vacant. It is clear that among all samples, the CuCoO_2_/ZnO (0.1 : 1) nanocomposite has the greatest dye loading. The amount of dye loading subsequently decreases as the amount of CuCoO_2_ nanoparticles increases. This result is explained by improvements in total dye loading, light absorption efficiency, and dye aggregation. In the end, inhibiting dye aggregation on nanocomposites is more effective at preventing dye adsorption than aggregating nanoparticles.

### 3.5. Photovoltaic, EIS, and IPCE studies

Several pastes having different mole ratios of CuCoO_2_/ZnO ((0.05, 0.1, 0.15, 0.2) : 1) were made in order to investigate the impact of CuCoO_2_ nanoparticles on the photovoltaic characteristics of the ZnO photoanodes in DSSCs. Energy efficiency was calculated according to: PCE= (*J*_sc_ × *V*_oc_ × FF)/*P*_in_, where *P*_in_ is the total amount of light radiated to the cell (100 mW cm^−2^, AM 1.5).^60^ The current density–voltage (*J*–*V*) curve for each cell can be employed to calculate *V*_oc_ and *J*_sc_. Fig. 11b displays the *J*–*V* curves of the DSSCs under illumination. The *J*_sc_, *V*_oc_, FF, and PCE data of the cells are summarized in Table 2. For the reference DSSC based on ZnO photoanode, the FF is determined to be 44.47% and the *V*_oc_ = 597.00 mV, *J*_sc_ = 11.51 mA cm^−2^, PCE = 3.05%. Among all of the cells, the ZnO cell has the lowest photovoltaic values. *V*_oc_, *J*_sc_, and PCE rise with increasing percentage of the CuCoO_2_ added to ZnO layer, achieving maximum values for the CuCoO_2_/ZnO (0.1 : 1) cell with *V*_oc_ = 687.84 mV, *J*_sc_ = 14.56 mA cm^−2^, highest PCE of 6.27%, and FF = 62.67%. Nevertheless, there is a minor reduction in the photovoltaic characteristics when CuCoO_2_ content reaches 0.2 (CuCoO_2_/ZnO (0.2 : 1)) with *V*_oc_ = 641.37 mV, *J*_sc_ = 11.81 mA cm^−2^, PCE = 4.25%, and FF = 56.61%. It is important to remember that the reason for gradual increase of *J*_sc_ in the CuCoO_2_/ZnO_2_ (0.1 : 1) cell compared to ZnO cell is because the photoanode's specific surface area has been increased (proved by BET analysis). Moreover, UV-visible tests on sensitized ZnO and various CuCoO_2_/ZnO ((0.05, 0.1, 0.15, 0.2) : 1) photoanodes have demonstrated that the absorbance increases with increasing CuCoO_2_ nanoparticle dosage up to CuCoO_2_/ZnO (0.1 : 1) content, demonstrating because of their greater specific surface areas, photoanodes with more nanoparticle contents loaded more dye molecules. Thus, a greater photocurrent is produced when more dye molecules are adsorbed.

**Table 2:** The *J*_sc_, *V*_oc_, FF, PCE, *R*_s_, *R*_1_, and *R*_2_ and *C*_2_ capacitance values for all assembled DSSCs

Photoanode	Parameter							
	*J*_sc_ (mA cm^−2^)	*V*_oc_ (mV)	FF (%)	PCE (%)	*R*_s_ (Ω cm^2^)	*R*_1_ (Ω cm^2^)	*R*_2_ (Ω cm^2^)	*C*_2_ (μF)
ZnO	11.51	597.00	44.47	3.05	2.94	13.31	59.23	268.69
CuCoO_2_/ZnO (0.05 : 1)	13.73	675.80	59.59	5.53	4.97	8.25	53.36	472.63
CuCoO_2_/ZnO (0.1 : 1)	14.56	687.84	62.67	6.27	4.61	6.43	50.06	503.85
CuCoO_2_/ZnO (0.15 : 1)	12.61	646.84	56.90	4.64	4.62	5.58	55.36	455.57
CuCoO_2_/ZnO (0.2 : 1)	11.81	641.37	56.61	4.25	4.00	10.02	57.32	440.05

**Fig. 12:**
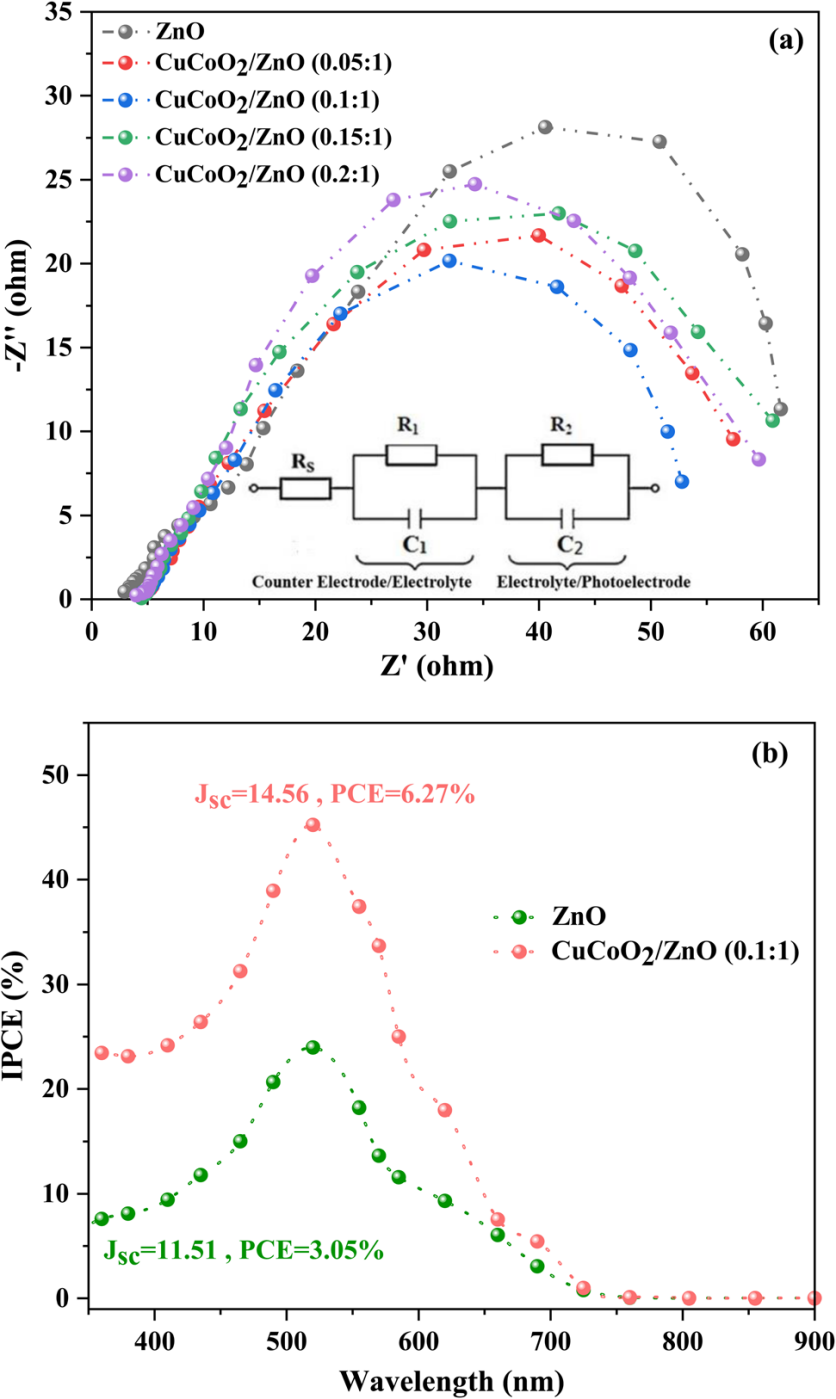
(a) The Nyquist diagrams of all solar cells assembled with ZnO and ZnO/CuCoO_2_ photoelectrodes. (b) IPCE spectra of DSSCs based on ZnO and CuCoO_2_/ZnO (0.1 : 1) photoelectrodes.

EIS studies on the cells at varied CuCoO_2_/ZnO ((0.05, 0.1, 0.15, and 0.2) : 1) ratios have been carried out in order to better understand the impact of CuCoO_2_ nanoparticle on ZnO-based DSSC operation in the presence of light at open-circuit voltage, between 1 and 10^5^ Hz. Impedance spectroscopy is a powerful method to determine the charge transfer resistance at the semiconductor/electrolyte interface.^61^ It has helped us to comprehend the characteristics of electron transport and the process of charge recombination.^62^Fig. 12a displays the Nyquist curves of the individual cells. Two semicircles with widths corresponding to the charge transfer resistance at the electrolyte/Pt-FTO interface (*R*_1_) and the charge recombination resistance at the CuCoO_2_/ZnO ((0, 0.05, 0.1, 0.15, 0.2) : 1)/dye/electrolyte interface (*R*_2_) are shown in the high and medium frequency regions, respectively. The series resistance (*R*_s_) of the cells is determined using the curve shift on the real impedance axis. *R*_s_ is mostly ascribed to the cell's electrical contacts, substrate resistance, electrolyte resistivity, and wiring.^63^ The parameters *R*_s_, *R*_1_, and *R*_2_ for each cell are displayed in Table 2. It is found out that the CuCoO_2_/ZnO (0.1 : 1) photoanode, which has the most porous surface of all the cells and the lowest resistance, also has the shortest semicircle radius, indicating it is the best photoelectrode material.

Table 2 indicates that the charge transfer resistance (*R*_1_) and charge recombination resistance (*R*_2_) decrease by adding CuCoO_2_ into the ZnO matrix. Notably, it seems that the *R*_1_ and *R*_2_ values of all devices are close to each other. This can be attributed to the addition of low CuCoO_2_ contents (0.05, 0.1, 0.15, 0.2 wt%) to the ZnO matrix. However, a comparison of the charge transfer resistance (*R*_1_) values of the photoanodes based on ZnO, CuCoO_2_/ZnO (0.1 : 1) and CuCoO_2_/ZnO (0.15 : 1) displays that the *R*_1_ has been decreased by 58 and 52%, respectively, in the composite photoanodes. This result confirms that the *R*_1_ value in the ZnO containing DSSC is very larger than those of the devices fabricated using CuCoO_2_/ZnO (0.1 : 1) and CuCoO_2_/ZnO (0.15 : 1) materials.

Among composite photoanodes, the lowest and the highest *R*_1_ values are measured for the DSSCs composed of CuCoO_2_/ZnO (0.15 : 1) and CuCoO_2_/ZnO (0.2 : 1) photoanodes, respectively, confirming the smallest charge transfer resistance is measured for the DSSC based on CuCoO_2_/ZnO (0.15 : 1) photoelectrode (5.58 Ω cm^2^). On the other hand, the *C*_2_ charge capacitance value of this device (455.57 μF) is lower than that of the DSSC with CuCoO_2_/ZnO (0.15 : 1) photoanode (503.85 μF). As the *R*_1_ and *R*_2_ values of the devices assembled based on CuCoO_2_/ZnO (0.10 : 1) and CuCoO_2_/ZnO (0.15 : 1) photoanodes are very close to each other, therefore, it can be found that the best performance of the champion DSSC with CuCoO_2_/ZnO (0.15 : 1) composite material is related to its low charge transfer resistance, suitably high charge recombination resistance, and the highest *C*_2_ charge capacitance.

IPCE spectroscopy was used to test the capacities of DSSCs to capture light. The IPCE spectra of ZnO and CuCoO_2_/ZnO ((0.1) : 1) based DSSCs are displayed in Fig. 12b. In the visible area, close to 520 nm, DSSCs exhibit a broad peak. Generally, improving the DSSC's light harvesting efficiency is another crucial element which influences its performance. The greater *J*_sc_ during short-circuit situation is indicated by the higher IPCE value.^64^ The greatest IPCE absorption at 520 nm achieved by the DSSCs based on the CuCoO_2_/ZnO (0.1 : 1) and ZnO photoanodes, respectively, are 45.22% and 23.95%. The strong scattering impact of the highly ordered CuCoO_2_/ZnO composite structure and the fine dispersion of CuCoO_2_ nanoparticles dispersed onto the ZnO particles with high specific area are responsible for this significant improvement in the IPCE.

## 4. Conclusion

For highly effective DSSCs, new and efficient photoelectrode materials must be developed. In the current work, a novel, cost-effective, feasible and effective DSSCs have been assembled using zinc oxide and copper based delafossite CuCoO_2_ (CuCoO_2_/ZnO) composites. ZIF-8 was heated to create the ZnO nanoparticles, which were then combined with the CuCoO_2_ material. In order to fabricate DSSC devices, CuCoO_2_/ZnO nanocomposite photoanodes, N719 dye, and Pt counter electrode were utilized. The FESEM images showed that the CuCoO_2_/ZnO nanocomposites formed uniform particles and the XRD data indicated the presence of polycrystalline ZnO nanoparticles. The existence of Cu, Co, Zn, O, and N was verified by EDX analysis, and UV-Vis DRS spectra revealed that CuCoO_2_/ZnO (0.1 : 1) absorbed the utmost light and had the lowest optical band gap (2.51 eV) energy. The surface area of the nanostructures increased as a result of the addition of CuCoO_2_ nanoparticles to ZnO, so that the highest surface area of 70.32 m^2^ g^−1^ was measured for the CuCoO_2_/ZnO (0.1 : 1). Moreover, the CuCoO_2_/ZnO (0.1 : 1) nanocomposite showed the highest dye loading among all the samples. According to photovoltaic studies, the DSSC with CuCoO_2_/ZnO (0.1 : 1) photoanode had the highest PCE of 6.27%, which was 2.05 times greater than that of the device based on the bare ZnO (3.05%). The CuCoO_2_/ZnO (0.1 : 1) composite's photovoltaic data were *J*_sc_ = 14.56 mA cm^−2^, *V*_oc_ = 687.84 mV, and FF = 62.67%, respectively. The CuCoO_2_/ZnO (0.1 : 1) composite electrode, with the most porous surface among all the samples and the lowest resistance, exhibited the smallest semicircle radius, according to the EIS analysis. The high photoconversion efficiency has been described in terms of pore size, surface area, and IPCE = 45.22%. The findings proved that CuCoO_2_/ZnO (0.1 : 1) composite performed the best among all other samples and therefore it would be a promising photoanode for DSSCs.

## Data availability

The data corresponding to this paper can be provided on request.

## Conflicts of interest

Authors declare that they do not have any conflicts of interest.

## Supplementary Material
